# Bioinformatics analysis of miRNAs in the neuroblastoma 11q-deleted region reveals a role of miR-548l in both 11q-deleted and *MYCN* amplified tumour cells

**DOI:** 10.1038/s41598-022-24140-6

**Published:** 2022-11-17

**Authors:** Sanja Jurcevic, Simon Keane, Emmy Borgmästars, Zelmina Lubovac-Pilav, Katarina Ejeskär

**Affiliations:** 1 School of Bioscience, Systems Biology Research Centre, University of Skövde, Skövde, Sweden; 2 School of Health Science, DHEAR, Translational Medicine, University of Skövde, Skövde, Sweden; 3 Department of Surgical and Perioperative Sciences/Surgery, Umeå University, Umeå, Sweden

**Keywords:** Cancer genetics, Biomarkers, Molecular biology, Non-coding RNAs

## Abstract

Neuroblastoma is a childhood tumour that is responsible for approximately 15% of all childhood cancer deaths. Neuroblastoma tumours with amplification of the oncogene *MYCN* are aggressive, however, another aggressive subgroup without *MYCN* amplification also exists; rather, they have a deleted region at chromosome arm 11q. Twenty-six miRNAs are located within the breakpoint region of chromosome 11q and have been checked for a possible involvement in development of neuroblastoma due to the genomic alteration. Target genes of these miRNAs are involved in pathways associated with cancer, including proliferation, apoptosis and DNA repair. We could show that miR-548l found within the 11q region is downregulated in neuroblastoma cell lines with 11q deletion or *MYCN* amplification. In addition, we showed that the restoration of miR-548l level in a neuroblastoma cell line led to a decreased proliferation of these cells as well as a decrease in the percentage of cells in the S phase. We also found that miR-548l overexpression suppressed cell viability and promoted apoptosis, while miR-548l knockdown promoted cell viability and inhibited apoptosis in neuroblastoma cells. Our results indicate that 11q-deleted neuroblastoma and *MYCN* amplified neuroblastoma coalesce by downregulating miR-548l.

## Introduction

Neuroblastoma (NB) is a heterogeneous complex cancer arising from the transient embryonic neural crest^1^. It is one of the most common extra cranial solid tumours found in children^2^. NB can also be defined by the international neuroblastoma risk group (INRG)^3^, suggesting that low and intermediate NB have a good prospect for treatment, whereas, high risk tumors are more difficult to treat^4^. This difficulty to treat results in a higher mortality. High risk NB tumors have specific genetic alterations and are highly aggressive. Two of the most prevalent forms of high risk NB are; amplification of the *MYCN* proto-oncogene^5^ and unbalanced 11q-deleted loss of heterozygosity (LOH) tumors^2^, which account for approximately 20% and 30% of all cases, respectively. Currently, these two are the most difficult subgroups to treat with current therapies. These two common aggressive NB tumor subtypes rarely occur together and are therefore usually considered to be mutually exclusive^6^. In the rare cases with both 11q deletion and *MYCN* amplification the extent of 11q deletion is often reported to be 11q21-11qter and when there is no *MYCN* amplification the deletion extends to 11q14.1-11qter^6^. Extensive investigation into 11q has occurred since the identification as a frequent alteration. Currently, no gene or genes in the 11q-region have been proven to be NB tumor suppressors, although there are a number of candidates. Previously identified candidate tumor suppressor genes on 11q include; *DLG2* (11q14.1)^7^, *ATM* (11q22.3)^8^, *CADM1* (11q23.3)^9^and *H2AFX* (11q23.3)^6^. Common to all of these candidate tumor suppressor genes (TSGs) is the lack of a second hit. Based on the Knudson two hit hypothesis, the lack of a second hit could indicate that gene dosing is involved.

MicroRNAs (miRNAs) are a class of short single-stranded RNA sequences that have a large impact on the regulation of the transcriptome^10,11^. It has been estimated that as many as 60% of all human genes may be regulated post-transcriptionally by miRNA^12^. The regulatory effects of miRNAs are exerted by binding to the 3’-untranslated region (UTR) of a target messenger RNA (mRNA) via base pairing. Due to the short base pairs between miRNA and target mRNA one miRNA can bind to several (up to hundreds of) transcripts and a single transcript can be targeted by several miRNAs^13,14^. MicroRNAs have been demonstrated to play a major role in a broad range of important biological processes such as development, differentiation, growth and metabolism^15^. It has been shown that aberrant expression of miRNAs correlates with certain features, such as tumorigenesis, differentiation status and clinical outcome^16^.

Several studies have shown that miRNAs can play a critical role in initiation and progression of cancer by acting as tumour suppressors or oncogenes^17^. The gain of additional copies of a chromosomal region harbouring miRNAs may lead to overexpression of these miRNAs, which will in turn result in excessive down-regulation of their target genes. In cases where the target gene function as a tumour suppressor, its down-regulation may result in increased tumour growth. Therefore, miRNA expression profiles can potentially be of great prognostic and diagnostic value and should be considered when trying to understand the complexity of neoplasia^18,19^. miR-21 is located on chromosome 17q21, which is amplified frequently in all subtypes of advanced-stage NB. Wang et al., show that by reducing the expression of miR-21 in NB cell lines, the expression of two tumour suppressor genes (*PDCD4* and *PTEN*) increases, leading to inhibition of cell proliferation and increased apoptosis in the NB cell line^20^. Conversely, the genetic alterations acquired during cancer development may include the loss of chromosomal regions harbouring miRNAs, and may therefore be one of the underlying reasons for the loss of growth control, since the targets of these miRNAs may include proto-oncogenes. For example, miR-193b has been implicated as causal in NB. Roth et al., have shown that overexpression of miR-193b in NB reduces cell growth via downregulation of the three oncogenes (*MCL1*, *Cyclin D* and *MYCN*). The results support the role of miR-193b as a potential therapeutic biomarker for NB^21^.

Multiple studies have reported that a common breakpoint region (11q14-qtel) exist in 11q-NB^6,7,22^. In this study, we aimed to identify miRNAs located in this region and their target genes that may be responsible for the development of NB.

## Results

### Bioinformatics analysis of miRNAs in the 11q14-qtel region and their target genes

**Figure 1:**
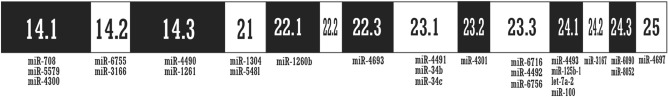
Map of chromosome 11q14-qtel with miRNA genes. The map shows the chromosomal positions of 26 miRNA genes within 11q14-qtel region.

To identify miRNAs at the chromosome 11 breakpoint regions we searched miRBase database and found 26 miRNAs within 11q14-qtel region (Fig. 1). We conducted a bioinformatics analysis of the miRNAs to find those with potential target genes that may be involved in development of NB.

**Figure 2:**
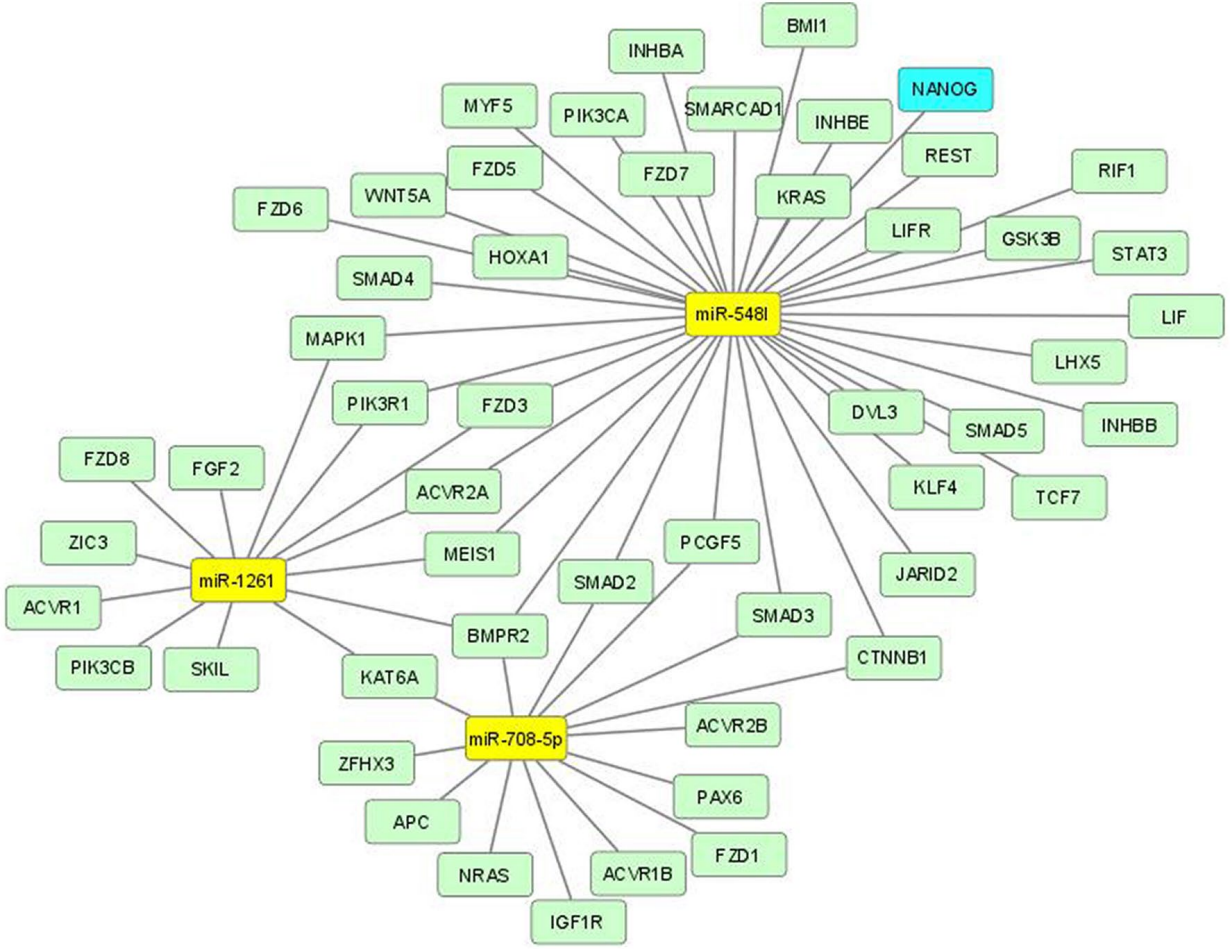
Network of miRNA-target mRNAs involved in signaling pathways regulating pluripotency of stem cells. The yellow box nodes represent miRNAs; green nodes describe target; blue box node represents hub gene.

Since miRNAs have important roles in various biological processes by post-transcriptional regulation of target mRNAs, we studied the function of the miRNAs by identifying putative target genes. The target genes of the 26 miRNAs were identified in silico, followed by a pathway analysis that was performed with the KEGG database to investigate the possible biological impact of the miRNAs and their target genes in development of NB. We found in total 61 KEGG pathways predicted to be influenced by these 26 miRNAs (Supplementary Table 1). As expected, target genes were enriched in pathways associated with cancer, including proliferation, evading apoptosis and DNA repair. We also noted that three miRNAs (miR-548l, miR-708-5p, and miR-1261) regulated 36% of all genes involved in signaling pathways regulating pluripotency of stem cells (Fig. 2).

**Figure 3:**
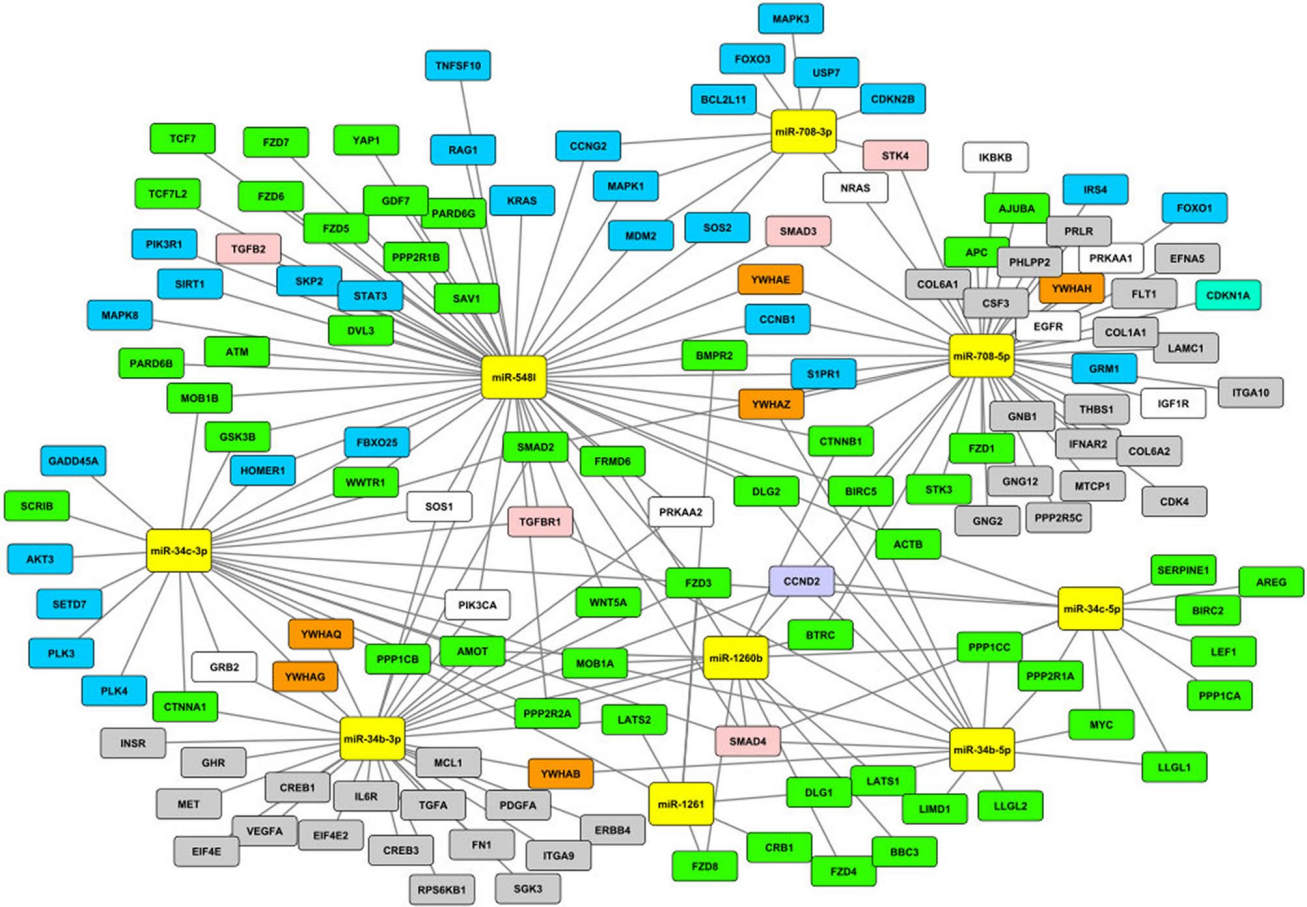
miRNA regulation of genes involved in Hippo, Foxo and PI3K-Akt pathway. The yellow box nodes represent miRNAs; green nodes describe genes located in the Hippo pathway; blue nodes describe genes located in the Foxo pathway; grey nodes describe genes located in the PI3K-Akt pathway; pink nodes describe genes located in both the Hippo and Foxo pathways; orange nodes describe genes located in both the Hippo and PI3K-Akt pathways; purple node describes gene located in the Hippo, PI3K-Akt and Foxo pathways; white nodes describe genes located in both the PI3K-Akt and Foxo pathways.

Results from KEGG analysis indicated that miR-548l was central, as it alone was predicted to be involved in 26 of 61 pathways and it regulated the central genes in several pathways, among those responsible for DNA repair, the Hippo, PI3K and Foxo signaling pathways (Fig. 3). Thus, miR-548l was chosen for further bioinformatics and experimental analysis in NB cells.

**Figure 4:**
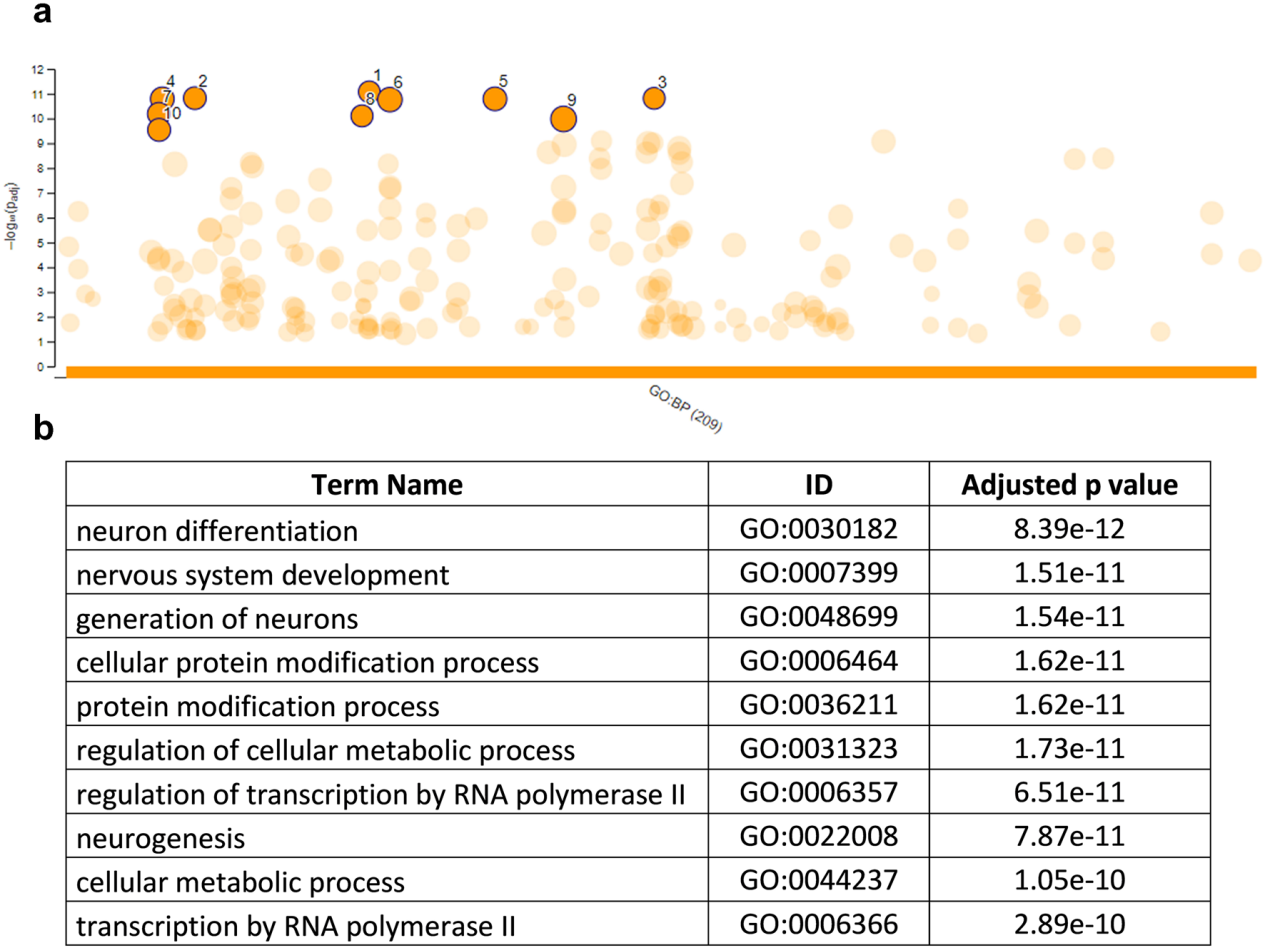
The 209 enriched biological processes controlled by miR-548l from at least 2 of the database lists. (**a**) the enriched biological processes of genes under the control of miR-548l (**b**) tabular result of the adjusted p values of 10 selected enriched terms. The data is presented as a Manhattan plot with the y axis representing the -log10 adjusted p value and GO ID number along the x axis, so that similar terms cluster on this plane. The functional enrichment analysis was performed using g:Profiler with g:SCS multiple testing correction method applying significance threshold of 0.05.

Gene ontology (GO) analysis of miR-548l target genes showed that target genes corresponding to the biological processes from GO categories were mainly involved in neuron differentiation, nervous system development, generation of neurons and neurogenesis (Fig. 4).

### Network analysis of protein targets for miR-548l

**Figure 5:**
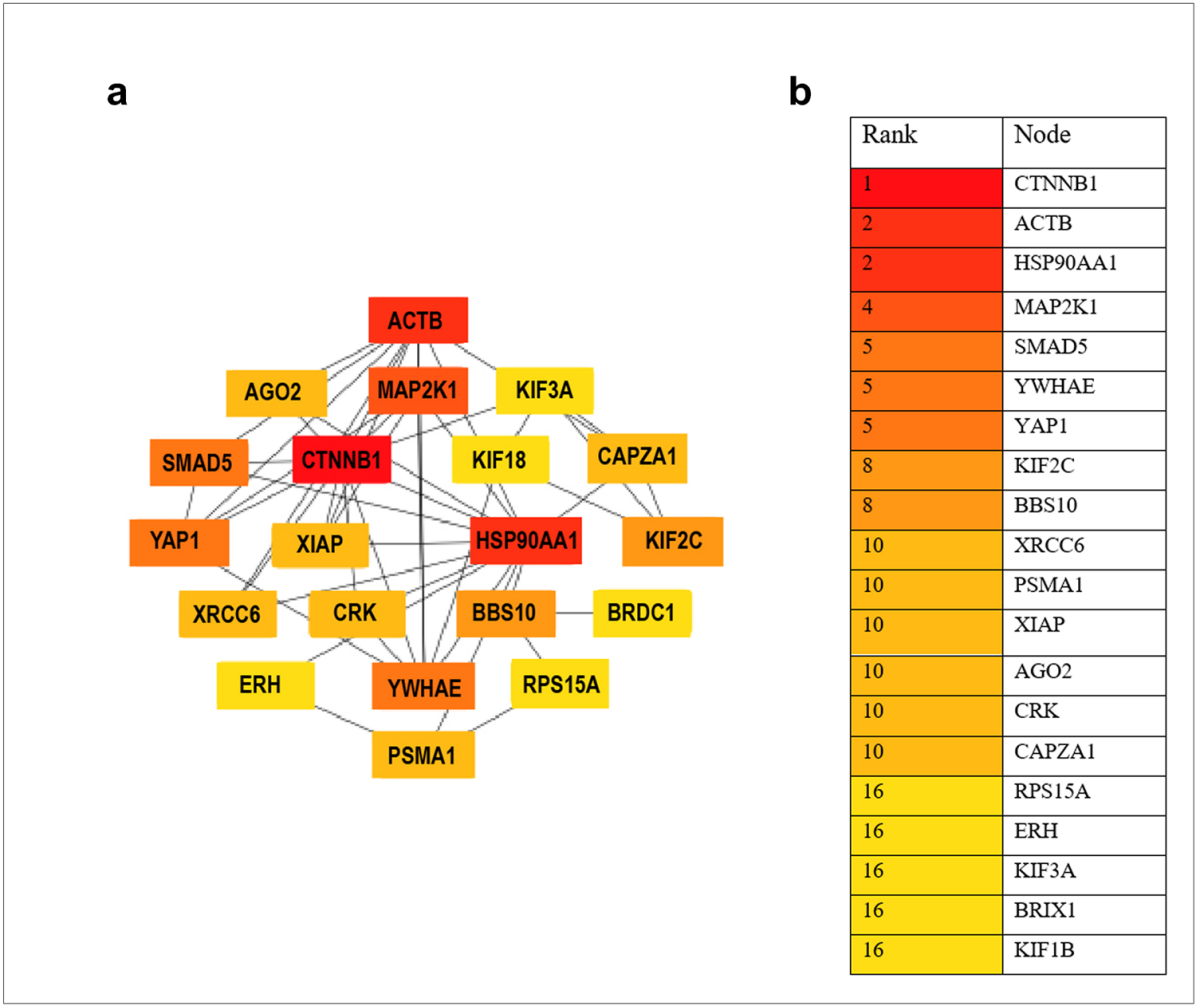
Hub genes among miR-548l target genes, (**a**) PPI network of the top 20 hub nodes targeted by miR548l. (**b**) Rank table for the top 20 hub nodes and their ranks based on degree algorithm. The darker red color, the higher the degree.

**Figure 6:**
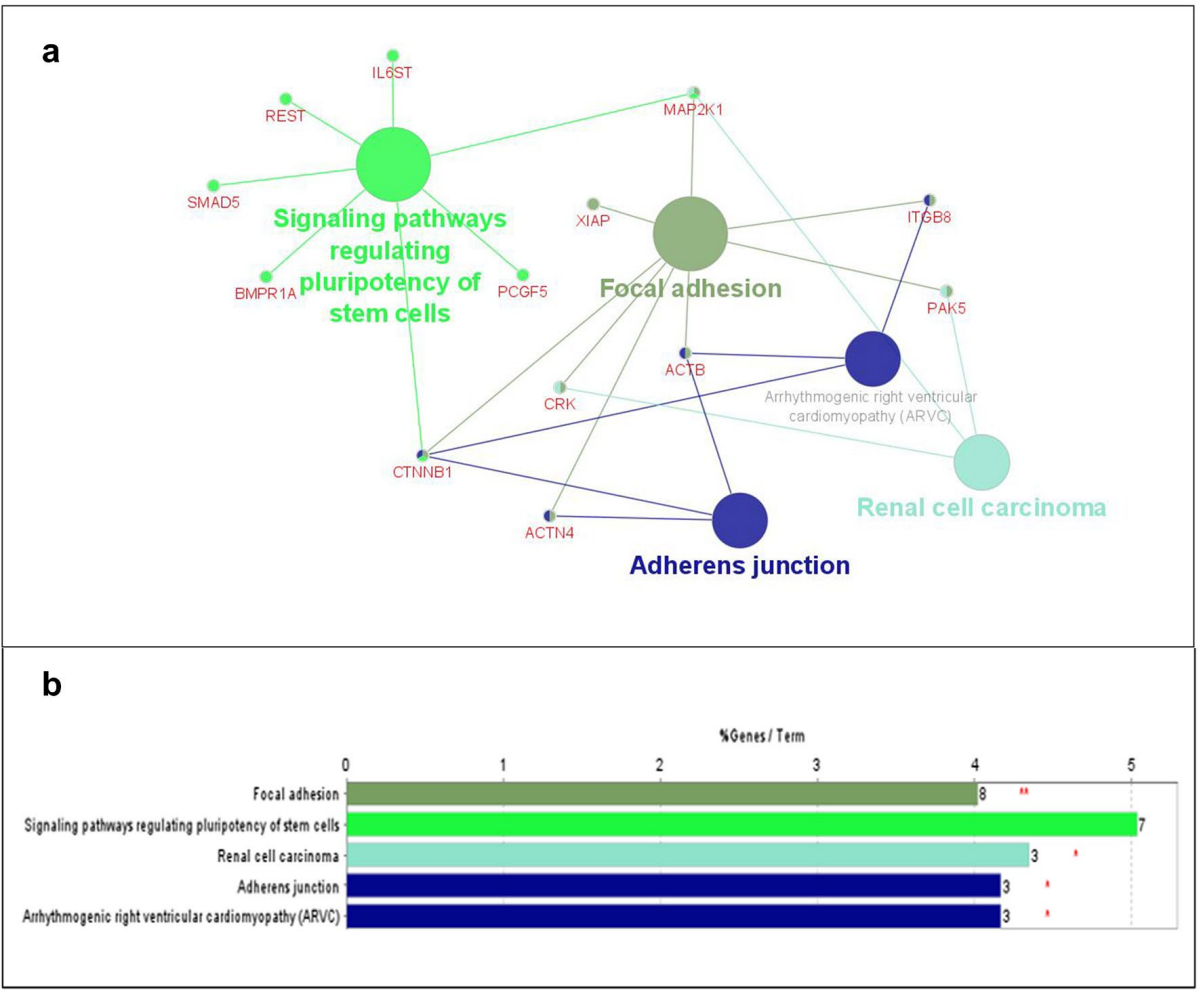
Enriched KEGG pathway for miR-548l target genes in ClueGO and CluePedia. (**a**) Enriched KEGG pathways for miR-548l targets. The node size represents the term enrichment significance (**b**) Bars represent the number of genes associated with each term/pathway. Percentage of genes per term is shown as bar label.

Additional network analysis and functional enrichment was performed for miR-548l targets to extract hub genes and pathways of potential relevance for disease. Figure 5a shows 20 top-ranked nodes based on Cytoscape plugin cytoHubba^23^ degree score. The hubs with the largest degree (≥ 10) are *CTNNB1*, *ACTB*, *HSP90AA1*, and *MAP2K1* (Fig. 5b). Cytoscape plugins ClueGO and CluePedia^24^ were used to identify enriched KEGG pathways as well as the interplay between these by showing genes that connect different pathways (Fig. 6, only pathways with p ≤ 0.05 are shown). *CTNNB1* was found to overlap between 3 significantly enriched KEGG pathways: adherens junction, focal adhesion and signaling pathways regulating pluripotency of stem cells (Fig. 6a).

Two of the significantly enriched pathways containing largest number of proteins that are targeted by miR-548l are focal adhesion (8% genes/term) and signaling pathways regulating pluripotency of stem cells (7% genes/term).

### miR-548l expression is repressed in high risk NB cells

**Figure 7:**
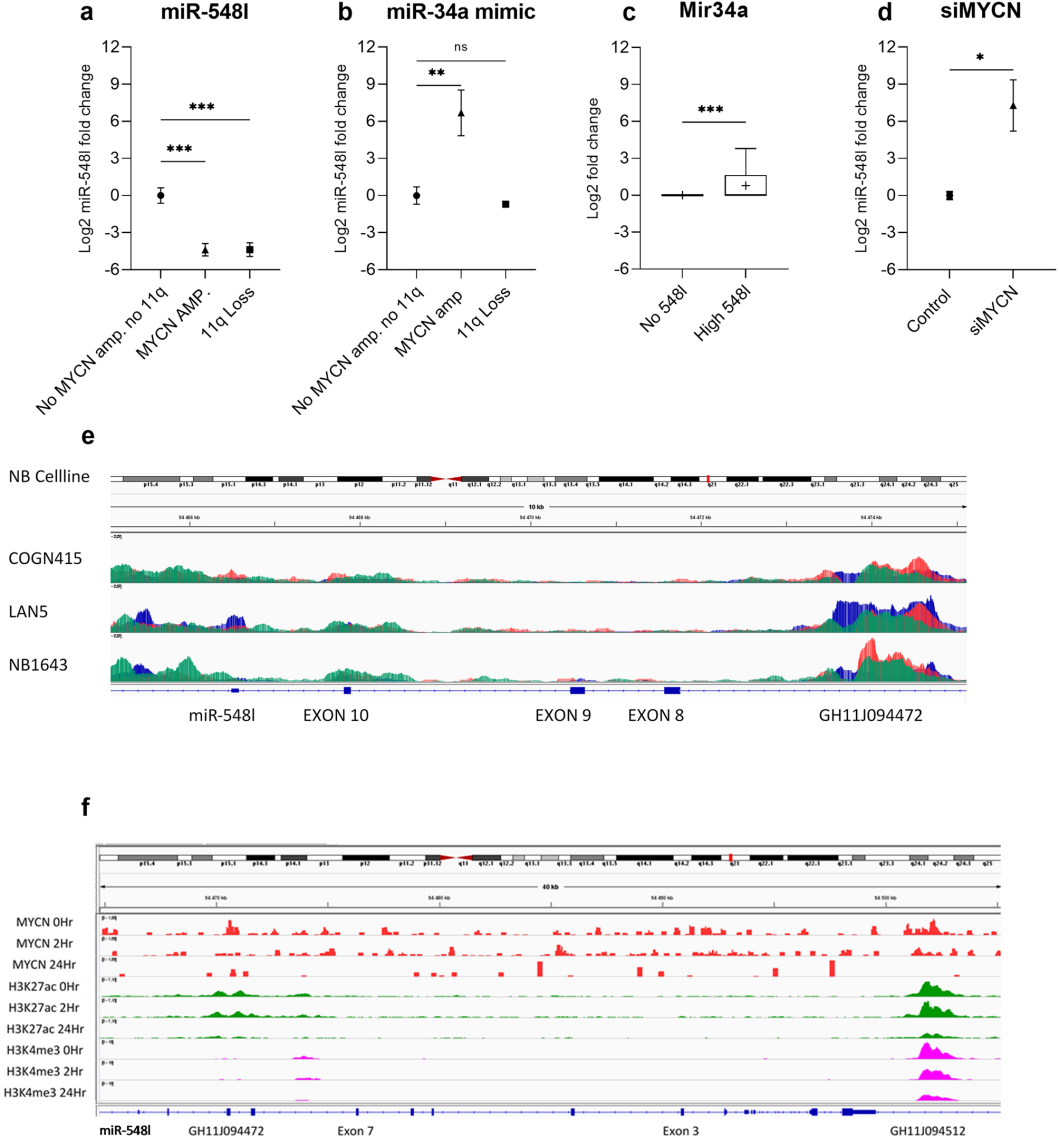
miR-548l is under the direct control of MYCN. (**a**) Expression of miR-548l in NB cells with 11q Deletion (Del)., MYCN amplification (amp.) or neither (Normal). (**b**) Expression of miR-548l expression quantified after miR-34a mimic expression in NB cells with 11q Deletion (Del)., MYCN amplification (amp.) or neither (Normal). (**c**) Expression of mir-34a in primary NB with no mir-548l expression compared to primary NB with high mir-548l expression (**d**) Expression of mir-548l after silencing of MYCN (siMYCN) compared to control in MYCN-amplified NB cells. (**e**) Open chromatin (ATAC-seq) overlay plots of MYCN amplified NB showing open chromatin (blue), MYCN binding (red) and H3K27ac (green) sequences 10 KB upstream of miR-548l. (**f**) The effect of MYCN silencing in SHEP-N21 cells over time. Chromatin binding (CHIP-seq) plots showing the binding of MYCN (red), H3K27ac (green) and H3K4me3 (pink) 40 KB upstream of miR-548l. The expression data are presented as log2 fold change and plotted as a scatter plot with a line at the mean. The CHIP-Seq and ATAC-Seq are expressed as rpm/bp. Statistical significance was determined by one-way ANOVA with a Holm-Šídák's multiple comparisons test*p < 0.05, **p < 0.01, ***p < 0.001, ns = not significant.

We evaluated the expression of miR-548l in NB cell lines with 11q deletion (del), *MYCN* amplification (amp.) or neither (no *MYCN* amp, no 11q del). We could show that there was a significantly lower expression in the *MYCN* amplified cells (log2 Fold Change (FC) = 4.38, p < 0.001) and in the 11q deleted cells (log2 FC = 4.37, p < 0.001) compared to the NB cell lines lacking either (Fig. 7a). To determine if the effect was as a direct result of *MYCN* amplification we transfected all three cell lines with miR-34a, a known inhibitor of *MYCN*. We found that miR-34a mimic transfection resulted in an increase in miR-548l expression in the *MYCN* amplified cells (log2 FC = 6.69, p < 0.001) when compared to the cells without *MYCN* amplification or 11q-deletion (Fig. 7b). There was no difference in miR-548l expression in the 11q deleted cells (log2 FC = -0.69, p = 0.66) compared to the cells without *MYCN* amplification or 11q deletion (Fig. 7b). We verified this correlation in a primary NB dataset, when comparing 33 NB:s without mir-548l expression to 51 NB:s with high mir-548l expression, we found very low expression of mir-34a in the NB:s lacking mir-548l (mean log2 FC = 0.00), while the NB:s with mir-548l expression had a higher mir-34a expression (mean log2 FC = 0.79; p < 0.001)(Fig. 7c). To further test if *MYCN* was directly responsible we silenced the expression of *MYCN* using siRNA targeted to *MYCN* in the *MYCN* amplified cell lines and detected an increase in miR-548l expression compared to control (log2 FC = 7.52, p < 0.05) (Fig. 7d). We subsequently investigated the mechanism to which *MYCN* could repress miR-548l expression. We used publicly available ATAC-Seq and CHIP-Seq data (GSE138315)^25,26^ to investigate the presence of; open chromatin, *MYCN* and *H3K27Ac* for 3 *MYCN* amplified NB cell lines, scaled to 10 KB. We could show that *MYCN* is bound to the enhancer GH11J094472 which controls miR-548l, with the presence of the *H3K27Ac* mark consistent across the 3 cell lines (Fig. 7e). The effect of *MYCN* silencing was determined using publicly available data showing the shutdown of *MYCN* over three timepoints (0 h, 2 h and 24 h) in SHEP21N cells (GSE80154)^27^. Here we could visualize the 80 KB upstream region of the miR-548l gene including the *MRE11* promoter GH11J094512 which is known to be under direct control of *MYCN*^28^*. MYCN* vacated the GH11J094472 promoter after 2 h indicating weak affinity with the *H3K27Ac* mark decreasing over time. The GH11J094512 promoter showed gradual decreases in bound *MYCN*, *H3K27Ac* and *H3K4me3* over time (Fig. 7f).

### Overexpression of miR-548l slowed proliferation and promoted apoptosis

**Figure 8:**
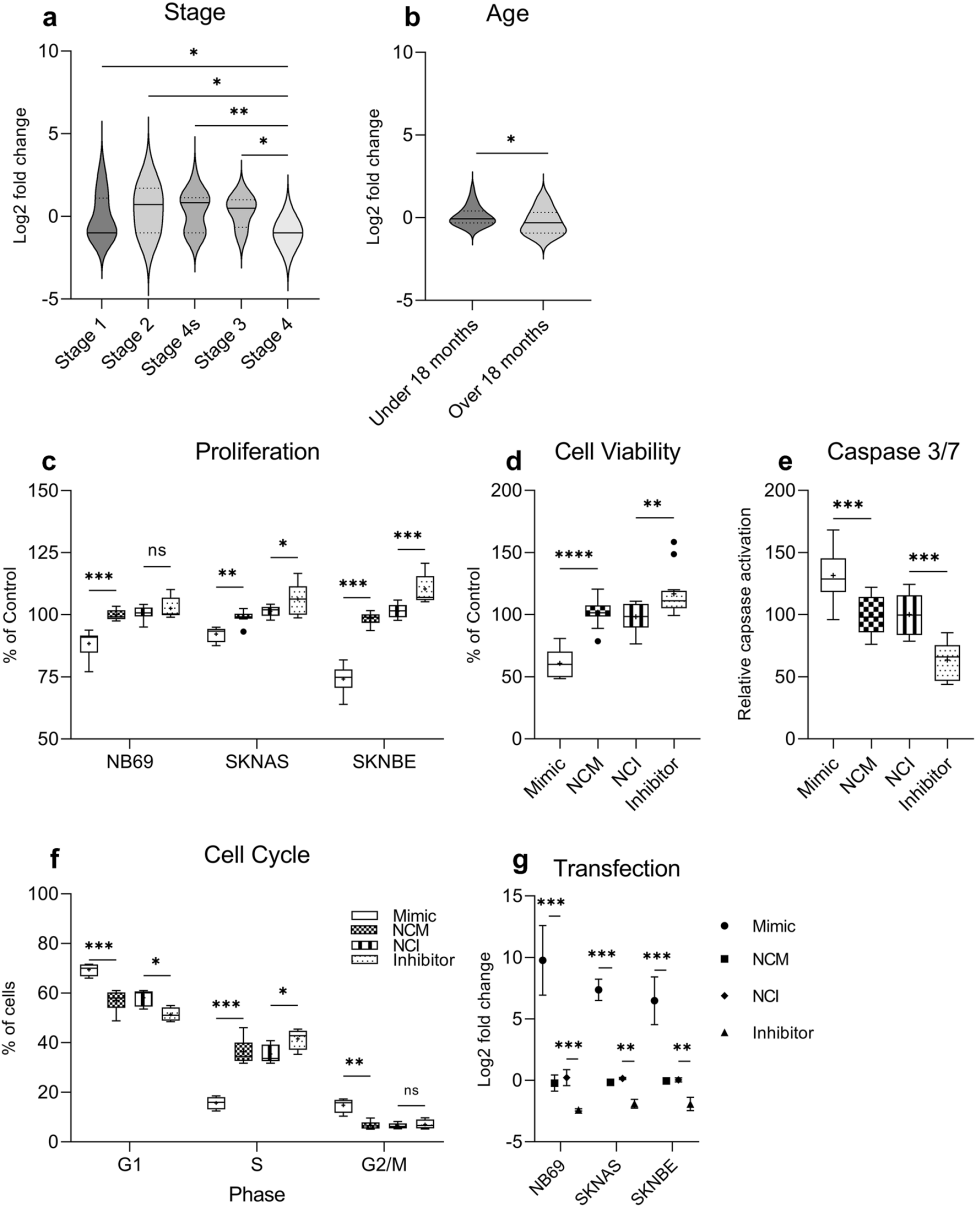
miR-548l expression in NB primary dataset GSE155945 stratified by (**a**) INSS stage and (**b**) age at diagnosis. Violin plots are presented with a slid line at the median and a dashed line showing the quartiles. The effect of miR-548l over expression (mimic) or miR-548l silencing (inhibitor) compared to negative control mimic (NCM) and negative control inhibitor (NCI) was determined in NB69, SKNAS (11q deletion) and SKNBE2 (*MYCN* amplified) cell lines regarding (**c**) cell proliferation, (**d**) cell viability (**e**) apoptosis initiation measured by caspase 3/7 activity. (**f**) Cell cycle analysis in response to miR-548I mimic or inhibitor in SKNBE2 cells. (**g**) miR-548l expression in NB69, SKNAS (11q deletion) and SKNBE2 (*MYCN* amplified) cell lines after transfection of miR-548l mimic, miR-548l inhibitor, negative control mimic (NCM) and negative control inhibitor (NCI). Boxplots are presented with a line at the median, and ‘ + ’ at the mean. The whiskers represent the 10th to 90th percentiles (Tukeys). The expression and protein data are presented as log2 fold change and plotted as a scatter plot with a line at the mean. Statistical significance was determined by one-way ANOVA with a Holm-Šídák's multiple comparisons test for groups of 3 or more or a paired t-test for exactly 2 groups. *p < 0.05, **p < 0.01, ***p < 0.001, ****p < 0.0001, ns = not significant.

WE used publicly available data to investigate miR-548l expression based on INSS stage and age at diagnosis (GSE155945)^29^. We found that miR-548l expression in NB was low in stage 4 and in patients of older age (Fig. 8a,b).

We next determined the effects on proliferation of miR-548l overexpression or silencing. Overexpression of miR-548l in NB cells (mir-548l mimic) resulted in slower proliferation compared to the negative control mimic (NCM) for NB69, SKNAS and SKNBE cells; 11.85%, 7.92% and 25.78% respectively, p < 0.001 (Fig. 8c). Inhibition of miR-548l increased proliferation in SKNAS and SKNBE by 6.15% and 10.4% respectively compared to negative control inhibitor (NCI), p < 0.001, with no change in NB69 (Fig. 8c). MiR-548l inhibitor promoted the cell viability of SKNBE cells as compared to NCI (19.0% increase, p < 0.01, Fig. 8d), while miR-548l mimic decreased the viability of SKNBE cells compared to NCM (40.2% decrease, p < 0.0001, Fig. 8d). We also found that the activity of caspase 3/7, the effector of apoptotic pathway, was increased by miR-548l mimic (31.4% increase, p < 0.001) and inhibited by miR-548l inhibitor (36.4% decrease, p < 0.001) in comparison with mimic- and inhibitor negative controls (Fig. 8e). We subsequently measured the cell cycle state of SKNBE cells after miR-548l overexpression or silencing. We could show that the cells accumulated in the G1 (12.5%, p < 0.001) and G2/M (8.1%, p < 0.001) phase, with fewer cells in the S Phase after miR-548l overexpression (mimic) (20.59%, p < 0.001) (Fig. 8f). Inhibition of miR-548l expression resulted in a decrease in the number of cells in G1 phase (5.5%, p < 0.05) and an associated increase in S phase cells (5.2%, p < 0.05). No difference in G2/M was detected with inhibition of miR-548l (Fig. 8f). The significant silencing (on average twofold, p < 0.01) or upregulation (on average eightfold, p < 0.001) of miR-548l after inhibitor or mimic transfections compared to NCM and NCI were confirmed by qPCR, for NB69, SKNAS and SKNBE cells (Fig. 8g).

## Discussion

NB is a childhood tumor that is responsible for approximately 15% of all childhood cancer deaths. While survival from various pediatric tumors has increased dramatically, survival of patients with NB continues to be poor. The subgroup of NB with deletion of chromosome arm 11q^30^ are highly aggressive and one of the most difficult subgroups to treat with current therapies^31^ with low overall survival^30^. Multiple studies have reported that a common breakpoint region (11q14-qtel) exist in high stage NB^6,7,22^. The mutual exclusivity of the 11q loss and *MYCN* amplification, despite themselves been common alteration in high risk NB indicates that they have a similar mechanism of action.

We identified and selected targets of the twenty-six miRNAs identified with the 11q14-qtel region and established their roles using biological process via KEGG pathway analysis. The biological pathways that were enriched included several cancer related pathways including; Hippo, PI3K and Foxo pathways (Fig. 3). Hippo signaling has been shown to regulate cell proliferation, neurogenesis and apoptosis^32^. We subsequently selected miR-548l for further investigation as it was expressed in neural tissues, and bioinformatics analysis revealed interesting target genes. We could show that there was a decrease in proliferation of NB cells after the restoration of miR-548l expression as well as a decrease in the percentage of cells in the S phase (Fig. 8). When combined with the results of the target genes using G:profiler we showed an enrichment of the genes associated with the development of the nervous system (Fig. 7) including; neurogenesis and neuron differentiation and neuron development all of which are altered in high risk NB^33^ and consistent with the function of Hippo signaling. Both miR-548l silencing an upregulation caused significant changes in the miR-548l level, which led to alterations in NB cell viability and apoptosis. Our study shows that six miRNAs from 11q14-qtel region regulate 45% of the genes that are involved in the Hippo signaling pathway (Fig. 3). The Hippo pathway gene, *YAP* (also known as *YAP1*) is regulated by miR-548l. Amplification of the *YAP* gene has been reported in a variety of tumors, including medulloblastomas^34^, prostate^35^ and colorectal cancer^36^ and *YAP* has been identified as a novel regulator of NB proliferation, tumorigenesis and invasion^37^.

The PI3K-Akt^38^ and Foxo^39^ pathways are also important in maintaining genomic stability in response to DNA damage, with Foxo overexpression in NB predicting adverse clinical outcomes^40^. The PI3K-Akt pathway was enriched as about 15% of the genes in this pathway were targets of miR-708-3p and miR-34b-3p (p < 0.01, Supplementary Table 1). Our analysis shows that the set of 52 genes implicated in the PI3K-Akt pathway are potential targets for miR-708-5p and miR-34b-3p.

*CTNNB1* was found to overlap between 3 significantly enriched KEGG pathways: adherens junction, focal adhesion and signaling pathways regulating pluripotency of stem cells (Fig. 6a). The *CTNNB1* encoded protein, β-catenin, has been shown to be highly expressed with aberrant localization in non-*MYCN* amplified high risk tumors^41^. Additionally, high expression of β-catenin has been shown to reduce cellular sensitivity to the ALK inhibitor crizotinib^42^. Among other top ranked hubs, *HSP90AA1* has been directly associated with NB in a previous study, showing that *Hsp90* inhibition in NB cell lines led to significant growth suppression, a decrease in *MYC* and *MYCN* expression, and an increase in protein expression of *p53*^43^*.* Furthermore, it has been shown to be associated with cisplatin resistance in NBs^44^. In previous work it was also shown that one of our highly ranked hubs, *YAP1*, promotes the proliferation of NB cells^28^.

We have shown that miR-548l is predicted to interact with both *DLG2* (11q14.1) and *ATM* (11q22.3), two candidate TSGs in the 11q14.1-ter breakpoint (Fig. 3). As we have previously stated, all of the current candidate TSGs within this region lack a second hit, based on the Knudson two hit hypothesis. The interaction of a miRNA with a transcript often results in translational repression of the target sequence. However, this is not always the case as under specific circumstances a miRNA can promote gene expression^45^. The presence of two major transcriptional isoforms of *DLG2,* each with a unique 3´UTR region does provide a mechanism for miRNA induced differential expression. Previously established results, showed differential *DLG2* isoform expression in high INSS staged neuroblastoma tumors^46^, and we could also see a lower expression of mir548l in stage 4 primary neuroblastomas (Fig. 8), similar to the *DLG2* expression. *ATM* has previously been shown to act the DNA damage response pathway leading to intra S phase arrest and activation of the G2/M cell cycle checkpoint^47^, something that we show is activated in response to the mimic of miR-548l (Fig. 8). We could also see an effect of mir-548l expression on apoptosis signaling, which could be causative to the changes in cell proliferation seen in NB cells after mir-548l silencing or upregulation (Fig. 8).

Taken together, the heterozygous loss of 11q14.1-ter has been shown to result in alterations to miR-548l (Fig. 7), *DLG2*^7^ and *ATM*^48^ expression. The potential for interactions between miR-548l, *DLG2* and *ATM* is an interesting foundation for a cluster of genes on 11q coalescing within similar pathways. Further investigation into the additional effects of miR-548l on these genes is certainly warranted.

In this study we analyzed miRNAs from the 11q14.1-ter breakpoint, aiming to discover potential biomarkers for NB. We could show that 11q-deleted and NB *MYCN* amplified NB coalesce by downregulating miR-548l.

It is important to stress that bioinformatics approaches for miRNA target prediction and enrichment that were conducted in this study, although highly beneficial for explorative research, suffer from well-known drawbacks, such as lack of sensitivity and specificity. Future work needs to include more functional validation to future strengthen our findings and provide evidence for miR-548l potential role in disease pathogenesis and therapy, but it out of scope for this study.

## Methods

### Bioinformatics analysis of miRNAs in the 11q14-qtel region and their target genes

In order to identify miRNAs located at the chromosome 11 breakpoints, we used miRBase (release 22.1). MicroRNA target genes were collected from two sources; experimentally validated targets and computational predicted targets. Validated miRNA target genes were generated from TarBase (Version 8)^49^, which provides curated experimentally validated miRNA target genes from published literature. TargetScan (release 7.2)^50^ and microT (Version 5)^51^ algorithms were used to receive the predicted target genes. Both algorithms use miRNA sequence and 3’UTR of potential target mRNA as input files in FASTA format and determine their binding ability by calculating the minimum free energy for hybridization. Pathway analysis was also performed on miRNA target genes based on the KEGG database (http://www.genome.jp/keg/)52. The count number larger than 4 and FDR less than 0.01 were chosen as cut-off criterion. Determination and visualization of the enriched biological processes was performed using g:Profiler using the previously established gene lists ^53^. Analysis of hubs in network of proteins targets for miR-548l was performed with Cytoscape plugin cytoHubba^23^ (version 0.1). Plugins ClueGO (version 2.5.8) and CluePedia^24^ (version 1.5.8) were used to identify enriched KEGG pathways among miR-548l targets.

### Publicly available data

We used GSE138315 and GSE80154 from Gene Expression Omnibus (GEO; https://www.ncbi.nlm.nih.gov/geo/). ChIP-seq for MYCN, as well as ATAC-seq was performed in various neuroblastoma cell lines (GSE138315 and GSE80154). Data for analyses and comparison of miR-548l expression between the different patient subgroups for the miRNA dataset GSE155945 was imported from the R2 platform (http://r2.amc.nl).

### Cell lines and tissue culture

Human NB cell lines SHSY-5y (no MYCN, no 11q deletion), IMR32 (MYCN amplification), SKNAS (11q deletion), SKNBE (2) (MYCN amplification), and NB69 (no MYCN, no 11q deletion) were obtained from the ATCC Cell Line Collection (ATCC). SHSY-5y, IMR32, SKNAS and SKNBE (2) were maintained in RPMI 1640 culture medium supplemented with 10% FBS, 1% L-Glutamine, 1% HEPES solution, and 1% sodium pyruvate. NB69 was cultured in RPMI 1640 medium supplemented with 15% FBS, 1% L-Glutamine, 1% HEPES solution, and 1% sodium pyruvate. The cells were cultured in a humidified incubator at 37 °C with 5% CO_2_.

### Transfections

Cell lines were grown to 80% confluence and subsequently transfected with 5 pmol *mir*Vana® miRNA corresponding to; hsa-miR-548l Mimic (4,464,066, Thermo Fischer Scientific), hsa-miR-548l Inhibitor (4,464,084, Thermo Fischer Scientific), hsa-miR-34a-5p Mimic (4,464,066, Thermo Fischer Scientific), *mir*Vana™ miRNA Mimic, Negative Control #1 (4,464,058, Thermo Fischer Scientific) or *mir*Vana™ miRNA Inhibitor, Negative Control #1 (4,464,079, Thermo Fischer Scientific) using Lipofectamine™ RNAiMAX Transfection Reagent (13,778,030, Invitrogen) and subsequently harvested 48 h post transfection. Cell lines with *MYCN* amplification were grown to 80% confluence and transfected with either si*MYCN* or scrambled control and harvested 48 h post transfection. In brief, 30 pmol siRNA was complexed with 12 μl Lipofectamine RNAiMAX (13,778,030, Invitrogen) according to the manufacturer’s protocol (Thermo Fisher Scientific).

### Functional assays in tissue culture

100 µl cell suspension of SKNAS, SKNBE2 and NB69 (1 × 10^4^ cells/well) was seeded in 96-well culture plates (Corning Incorporated). After culturing to 80% confluence the supernatant was removed and transfection media was added to the cells. 48 h post transfection, cell proliferation was measured using the MTS/MPS Cell Titer 96® AQ_ueous_ One solution Cell Proliferation Assay (MTS) (G3582, Promega) and detecting the color variation (FLUOstar Omega, BMG Labtech) as per the manufacturer’s recommendations. The absorbance values were normalized to the mock transfection and expressed as a percentage. All experiments were repeated three times.

For the cell viability imaging assays, cells were stained with ready probes cell viability imaging kit (blue/green) (R37111, Invitrogen) or Cell event Caspase 3/7 staining kit (R37609; Invitrogen), according to the manufacturer’s instructions and imaged on EVOS M7000 to determine whether they were live (blue) or dead (green) or had active caspase 3/7 (green). The result was subsequently analyzed using EVOS analysis software (Version 1.4.998.659) to determine cell number. The relative number of cells was subsequently determined by normalizing against the control transfections.

Cell cycle analysis was performed using the Cell-clock cell cycle assay (C1000, Biocolor). Images were subsequently analyzed using ImageJ image analysis as per the manufacturer’s instructions. The data presented is the average of three biological replicates. Each experiment series was repeated in triplicate.

### Quantitative PCR (qPCR) analysis

The total RNA was extracted using the *mir*Vana™ miRNA Isolation Kit (AM1560, Thermo Fisher Scientific) and reverse transcribed in a 2-step process with TaqMan MicroRNA Assays Reverse Transcription Kit (4,366,597, Applied Biosystems). miRNA expression was quantified using hsa-miR-548l (002,904, Thermo Fischer Scientific), or hsa-miR-34a-5p (478,048, Thermo Fischer Scientific) and normalized to RNU6B (001,093, Thermo Fischer Scientific) using the Livak method^54^. RNA from transfected NB cell lines was extracted using the RNeasy plus mini kit® (Qiagen) according to the manufacturer’s protocol. The RNA concentration was quantified by NanoDrop (NanoDrop Technologies) and 10 ng of RNA was reverse transcribed into double stranded cDNA on a T-professional Basic Gradient thermal cycler (Biometra) using the TaqMan® MicroRNA Reverse Transcription Kit (4,366,596, Applied Biosystems). cDNA corresponding to 20 ng of RNA for each qPCR reaction was used. qPCR was performed on a Pikoreal qPCR System (Thermo Fisher Scientific) in triplicate.

### Statistical analysis

Data presented are plotted as either mean ± SD, Tukeys box and whisker plots showing IQR, line at the median, + at the mean with whiskers ± 1.5-fold of interquartile range or scatter plot from at least 3 independent experiments. For all multi-group analyses, differences were determined by one-way ANOVA test followed by Holm-Šídák's multiple comparison test. For comparisons between two unpaired groups a Mann–Whitney U test was used. For comparisons between paired samples a paired t-test was used: with a p value < 0.05 considered significant. *p < 0.05, **p < 0.01, ***p < 0.001. All analyses were conducted using GraphPad Prism version 9.2.0 for Windows, (GraphPad Software, www.graphpad.com).

## Supplementary Information

Supplementary Information.

## Data Availability

The datasets used and/or analysed during the current study available from the corresponding author on reasonable request.
